# B-cell lymphomas involving Waldeyer's ring characterized by distinctive clinical and histopathological features: a comparison of pediatric to adult patients

**DOI:** 10.18632/oncotarget.14581

**Published:** 2017-01-10

**Authors:** Lei Chen, Lika'a Fasih Y Al-Kzayer, Yi Liu, Tingting Liu

**Affiliations:** ^1^ Department of Pathology, Xinhua Hospital, Shanghai Jiaotong University School of Medicine, Shanghai, China; ^2^ Department of Pediatrics, Shinshu University School of Medicine, Matsumoto, Nagano, Japan; ^3^ Department of Pediatric Hematology/Oncology, Xinhua Hospital, Shanghai Jiaotong University School of Medicine, Shanghai, China

**Keywords:** B-cell lymphoma, Waldeyer's ring, MUM1 positive lymphoma

## Abstract

B-cell lymphomas involving Waldeyer's ring (WR) comprise heterogeneous histolopathological subtypes with a wide age range and distinctive clinical features. However, the comparison between pediatric and adult patients is scarce and limited in the literature. Thirty-three cases of B-cell lymphomas involving WR, were collected and evaluated by histolopathological, immunohistochemical and FISH analyses. The 33 cases were categorized into children and adolescents referred as pediatric group (*n* = 12), aged (3−19) years and the adult group (*n* = 21), aged (20−84) years. The pediatric group included Burkitt lymphoma (BL), diffuse large B-cell lymphoma (DLBCL) and MUM1-positive-lymphoma in 7, 3 and 2 cases, respectively. While the adult cases comprised of DLBCL, follicular lymphoma, and mucosa associated lymphoid tissue (MALT) lymphoma in 18, 2 and 1 case, respectively. Male gender was predominant in both groups with 3 folds frequency in the pediatric cases compared to 2 folds in the adults counterpart. Pediatric cases showed a significantly higher frequency of stage I disease (*P* = 0.019), germinal center B-cell (GCB) phenotype (*P* = 0.011), CD10-positive expression (*P* = 0.003), and MYC breaks (*P* = 0.029) compared to adults. However, MUM1 positive expression was less frequently detected in pediatric patients than adults (*P* = 0.082). BCL2 rearrangement was undetectable in both pediatric and adult groups. On the other hand, adult group had the significantly higher proportion of DLBCL (*P* < 0.001), BCL2 expression (*P* = 0.027) and stage II disease (*P* = 0.047) compared to pediatric group.

In conclusion, B-cell lymphomas involving WR presented with a wide age range, and evident variation in clinical features, histopathological subtypes and immunophenotypes between pediatric and adult age groups.

## INTRODUCTION

B-cell lymphomas involving Waldeyer's ring (WR) comprise heterogeneous histolopathological subtypes with a wide age range and distinctive clinical features. WR is composed of lymphoid tissue of the nasopharynx and oropharynx, including the adenoids (pharyngeal tonsil), the lymphoid tissue around the pharyngeal openings of the Eustachian tubes (tubal tonsils), the palatine tonsils, lymphoid tissue of the soft palate and base of the tongue (lingual tonsil) [1]. WR represents one of the most common extra nodal sites for lymphoma development [2]. The most common subtypes of Non-Hodgkin's lymphoma (NHL) involving WR, in children and adults, are Burkitt lymphoma (BL) and diffuse large B-cell lymphoma (DLBCL), respectively. Follicular lymphoma (FL) involving WR also occurs in both children and adults. Of note, DLBCL involving WR displays peculiar clinicopathological features compared to nodal counterpart [3]. Patients with a GCB subtype of DLBCL were shown to have a significantly better clinical outcome than those with the non-GCB subtype [4].

Pediatric FL (PFL), occurring rarely in pediatric and young adults, differs from usual adult FL (AFL) in their clinical, morphologic, immunophenotypic and molecular features [5]. Compared to nodal PFL, PFL in WR is characterized by the co-expression of IRF4/MUM1 and BCL6, in addition to the frequent expression of BCL2 in the absence of t(14;18), as well as the association with *IGH* gene rearrangement and *BCL6* gene alteration [6]. IRF4/MUM1-positive-lymphoma in WR of children and young adults has been recently considered a new separate entity [7].

The vast majority of NHLs involving WR are of B-cell origin with a wide age range. Given the available published literatures, adult cases were described in accordance to their distinctive features and found to be of high-grade histology, early stage disease and of favorable outcome, whereas reports concerned with pediatric patients were limited [8, 9]. Furthermore, comparison studies between the two age groups were scarce.

In this study, we summarized both the common and the different clinicopathological characters of pediatric and adult B-cell lymphomas involving WR. Moreover, we focused on MUM1 expression in variable histological subtypes of B-cell lymphomas involving WR in pediatrics as well as the adult patients.

## RESULTS

The clinical features of 36 cases of NHL involving WR, including 33 cases of B-cell lymphoma, 2 cases of NK/T-cell lymphoma and 1 case of T-cell lymphoblastic lymphoma, were summarized in Table 1.

**Table 1:** Clinical features of 36 cases with non-Hodgkin's lymphoma involving Waldeyer's ring

Case no.	Age (y)/ Gender	Extra nodal site	LN involvement	Stage	Treatment Protocol	Clinical follow-up (months)	Relapse status
**B-cell lymphoma**							
**1-BL**							
**1**	3/M	Nasopharynx and pancreatic gland	Cervical LN	III	CCCG-BNHL	30	No
**2**	3/M	Rt. palatine tonsil	Cervical LN	II	CCCG-BNHL	14	No
**3**	4/M	Nasopharynx, lung and T11 vertebrae	Cervical LN	III	CCCG-BNHL	12	No
**4**	4/F	Rt. palatine tonsil and Rt. parotid gland	Cervical, axillary, and inguinal LN	III	CCCG-BNHL	14	Yes
**5**	4/F	Rt. palatine tonsil	No involvement	I	NA	NA	NA
**6**	6/M	Nasopharynx and Rt. palatine tonsil	Cervical LN	II	CCCG-BNHL	20	No
**7**	6/M	Lt. palatine tonsil	Cervical LN	II	NA	NA	NA
**2-DLBCL**							
PDLBCL							
**8**	5/M	Rt. palatine tonsil	Cervical LN	II	NA	NA	NA
**9**	8/M	Lt. palatine tonsil	No involvement	I	CCCG-BNHL	19	No
**10**	19/F	Rt. palatine tonsil	No involvement	I	CCCG-BNHL	38	No
ADLBCL							
**11**	27/M	Lt. palatine tonsil	NA	NA	NA	NA	NA
**12**	42/M	Nasopharynx	NA	NA	NA	NA	NA
**13**	47/F	Bilateral palatine tonsil and nasopharynx	Cervical and abdominal LN	III	R-CHOP	14	No
**14**	48/F	Oropharynx, stomach, and spleen	Cervical and abdominal LN	III	R-CHOP+ R-EPOCH	13	No
**15**	53/M	Lt. palatine tonsil	NA	NA	NA	NA	NA
16*	54/M	Tonsil	Cervical LN	II	R-EPOCH	4	No
**17**	56/M	Rt. palatine tonsil	NA	NA	R-CHOP	18	No
**18**	57/M	Lt. palatine tonsil	Cervical LN	II	R-CHOP+ R-EPOCH	20	No
**19**	57/M	Nasopharynx and oropharynx	Cervical and axillary LN	II	R-CHOP +R-EPOCH	10	No
**20**	60/M	Lt. palatine tonsil	Cervical LN	II	NA	NA	NA
**21**	61/M	Lt. palatine tonsil	Cervical LN	II	R-CHOP	21	No
**22**	62/F	Rt. palatine tonsil	Cervical LN	II	R-CHOP	17	Yes
**23**	64/F	Nasopharynx	Cervical, axillary, and inguinal LN	III	R-CHOP	16	No
**24**	68/M	Rt. palatine tonsil and hypothyroid	Cervical LN	II	R-CHOP	32	No
25*	72/M	Rt. palatine tonsil	Cervical and inguinal LN	III	R-CHOP	8	No
**26**	84/M	Lt. palatine tonsil and parotid	Cervical and axillary LN	II	R-CHOP	6	No**
**27**	52/M	Lt. palatine tonsil	Cervical LN	II	R-CHOP	19	No
**28**	54/F	Oropharynx	Cervical LN	II	R-CHOP	3	No
**3- Follicular lymphoma**							
PFL (MUM1 positive lymphoma) *							
**29**	11/M	Nasopharynx and Lt. palatine tonsil	No involvement	I	CCCG-BNHL	26	No
**30**	7/M	Rt. palatine tonsil	No involvement	I	CCCG-BNHL	22	No
AFL / FL 3B							
**31**	64/F	Lt. palatine tonsil	Cervical LN	II	R-CHOP	17	No
**32**	60/F	Rt. palatine tonsil	Cervical LN	II	R-EPOCH	20	No
**4-MALT lymphoma**							
**33**	58/M	Oropharynx	No involvement	I	Radiation	8	No
**T-cell lymphoma**							
**NK/T-cell lymphoma**							
**34**	47/F	Nasopharynx	No involvement	I		NA	NA
**35**	44/M	Nasal cavity and nasopharynx	Cervical LN	II		12	Yes**
**T-Lymphoblastic lymphoma**							
**36**	3/M	Nasopharynx	Cervical LN	II		19	No

* In accordance to new classification “large B-cell lymphomas with *IRF*4 rearrangement”; ** Cases died from progressive diseases.

ADLBCL, adult diffuse large B-cell lymphoma; AFL, adult follicular lymphoma; BL, Burkitt lymphoma; DLBCL, diffuse large B-cell lymphoma; F, female; FL, follicular lymphoma; LN, lymph node; Lt., left; M, male; MALT, mucosa associated lymphoid tissue; NA, not available; PDLBCL, pediatric DLBCL; PFL, pediatric follicular lymphoma; R-CHOP, (R, Rituximab; CHOP, cyclophosphamide, adriamycin, vincristine, prednisone); R-EPOCH regimen (R, Rituximab; EPOCH, etoposide, prednisone, vincristine, cyclophosphamide, and adriamycin); Rt., right.

### Comparison of B-cell lymphoma between pediatric and adult groups of patients

**Figure 1:**
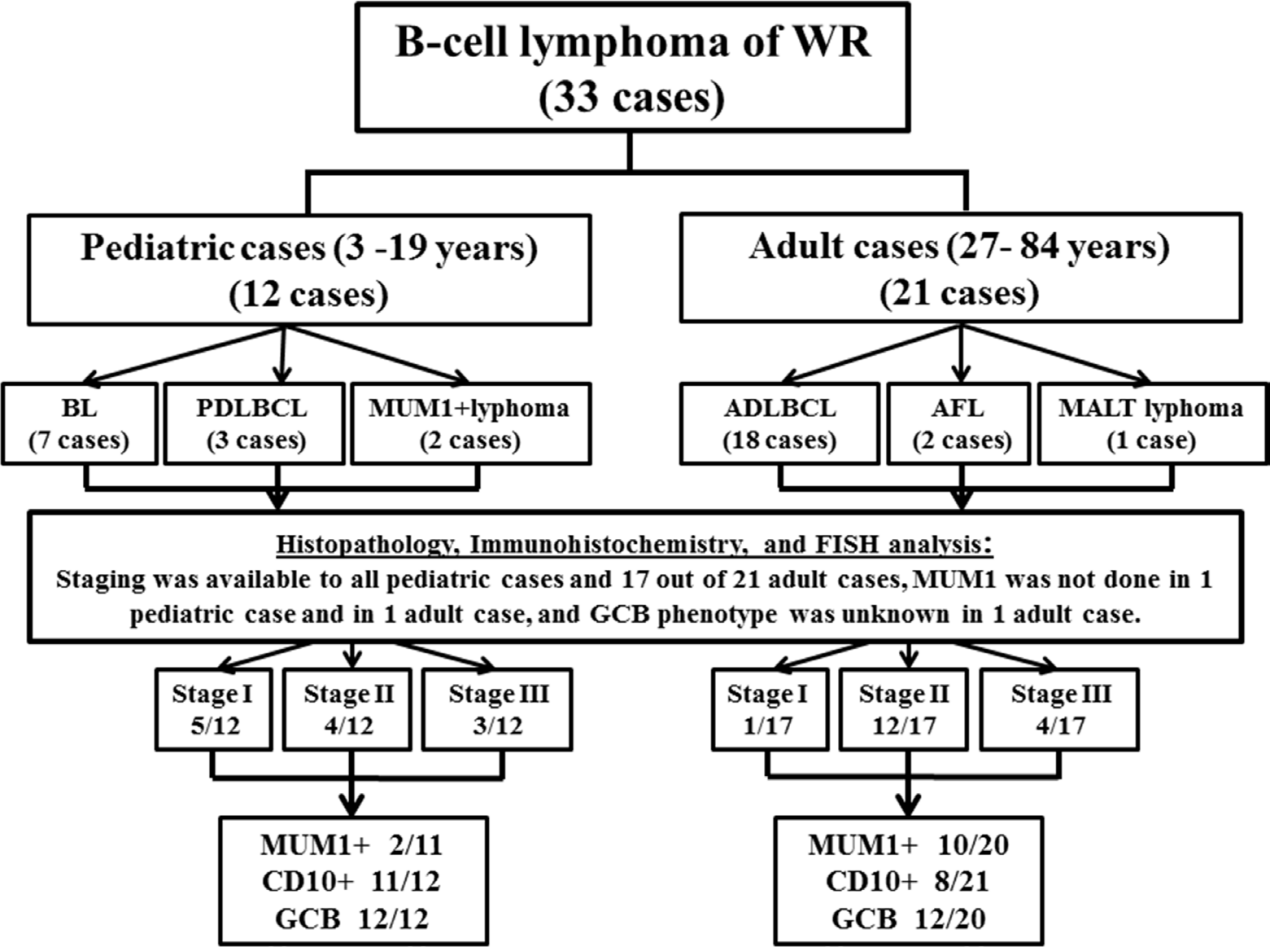
The description of the categorization of 33 cases of B-cell lymphoma of WR

The 33 patients with B-cell lymphoma, with a wide age range of 3–84 years (median, 52), were arbitrary classified as shown in Figure 1, into children and adolescent group (12 cases), aged 3–19 years (median, 5.5), hereafter referred to as pediatric group, and the adult group (21 cases) aged 27–84 years (median, 57). A male predominance (M: F = 23:10), was evident among the 33 patients, and the pediatric cases were 3 folds more frequent (M: F = 9:3), compared to 2 folds in adult cases (M: F = 14:7).

Symptoms were related to the location of the mass lesion, with more frequency of pharyngalgia and/or cervical lymphadenopathy. Pediatric patients exhibited other symptoms, including rhinorrhea, obstructive sleep apnea, and epistaxis, in addition to dysphagia and snoring. Given the available information about 29 out of 33 cases regarding disease stage, a high frequency was shown for the localized disease stages I and II, with 21% (6/29) and 55% (16/29) frequencies, respectively, compared to the advanced disease stage III, with a relatively lower frequency of 24% (7/29). Among the 12 pediatric patients, stage I, II and III were identified in 5 (42%), 4 (33%) and 3 (25%) cases, respectively. Compared with the group of 17 adults with the known staging, stage I, II and III, were identified in 1 (6%), 12 (70.5%) and 4 (23.5%), respectively. Thus, clinical stage I, was significantly more frequent among pediatric patients (*P* = 0.019), while on the contrary, stage II was significantly more common in the adult group (*P* = 0.047).

Data of the treatment and follow-up were available for 26 cases of B-cell lymphoma, including 9 pediatric and 17 adult patients. The 26 patients received surgical excision/biopsy, followed by chemotherapy (25 patient) or local radiation (1 patient). The median follow-up period was 17 months (ranging from 3 to 38 months) (Table 1). Two cases had relapsed including, one child with BL (case 4) and one adult patient with DLBCL (case 22). Meanwhile, case number 26 had developed multiple organ failure after the first cycle of chemotherapy and died.

**Figure 2:**
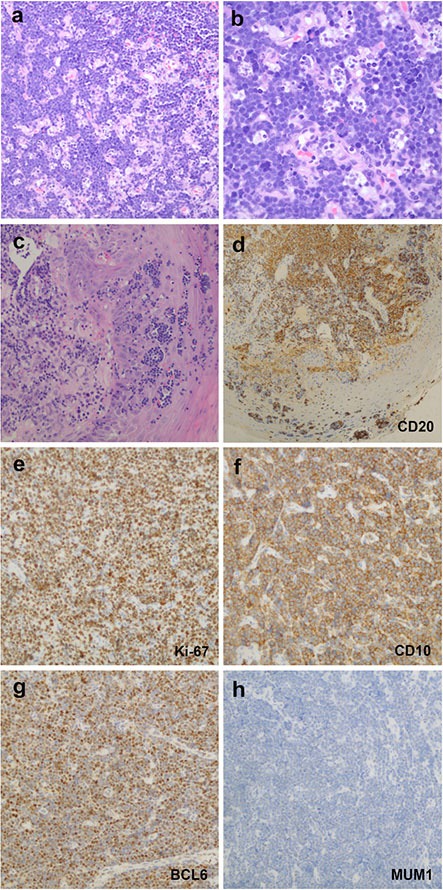
Histopathological and immunohistochemical characteristics of B-cell lymphomas involving WR (**A, B**), Case 1, BLs showed morphological features with a prominent starry-sky growth pattern at low magnification (A) Hematoxylin and eosin, HE, original magnification ×200) and a population of monomorphous medium-sized cells with round to slightly irregular nuclei, finely clumped chromatin and multiple small nucleoli at high magnification (B) HE, original magnification ×400). (**C–H**) Case 17, DLBCLs revealed that the neoplastic cells infiltrated the epithelium (Hematoxylin and eosin, HE, original magnification ×200). (D) Lymphoepithelial lesions showed positive expression for CD20 staining (HE, original magnification ×400). e, Ki-67 proliferation index was over 90% of neoplastic cells staining. The GCB subtype case showed positive expression of CD10 (F) and BCL6 (G) but a negative expression of MUM1 (H). Samples were stained for indicating target antigen by IHC; HE, original magnification ×200.

**Figure 3:**
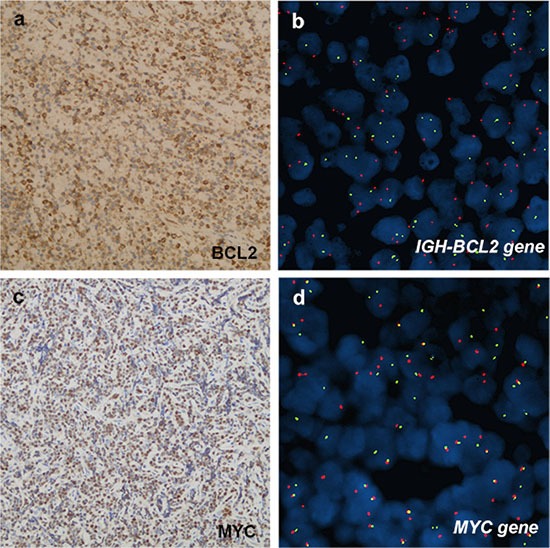
Immunohistochemical and cytogenetic features of B-cell lymphomas involving WR (**A, B**), case 8, PDLBCL. (A) BCL2 was positively expressed in neoplastic cells. b, FISH analysis did not detect a positive fusion signal in BCL2 of the samples. LSI IGH/BCL2 dual-color, dual-fusion probe signal pattern expected for t(14;18)(q32;q21). SpectrumOrange-labeled BCL2 probe and SpectrumGreen-labeled IGH probe correspond to 18q21 and 14q32, respectively. (**C, D**), case 3, BL. (C) MYC was positive expression. (D) Presence of LSIMYC Dual Color, BreakApart Rearrangement Probe signal pattern with abnormal nucleus showing a one orange, one green and one orange/green fusion signal pattern. Normal probe hybridizes to the band region 8q24. SpectrumOrange probe begins upstream of the 5′ end of MYC and SpectrumGreen probe starts downstream of 3′ of the MYC gene.

Immunophenotype study revealed that the 33 cases were consistently positive for B-cell markers (CD20, CD79a and PAX5) and variably positive for MUM1 and CD10 (Figure 2). As shown in Table 2, MUM1 was positive in 18% (2/11) pediatric cases, which were classified as MUM1-positive-lymphoma involving WR. MUM1 was also positive in 50% (10/20) adult patients, including 4 cases of GCB-DLBCL and 6 cases of non-GCB-DLBCL. The remaining patients were negative for MUM1, providing that it was not evaluated in 2 cases. CD10 was positive in 58% (19/33) cases, and it was significantly expressed in the pediatric group; 92% (11/12), compared to adult group; 38% (8/21), (*P* = 0.003). As shown in Figure 2 (F–H), 75% (24/32) of the cases presented with GCB phenotype and 25% (8/32) of cases were of non-GCB phenotype. GCB immunophenotype was the unique profile in the pediatric group; 100% (12/12), compared with the adult cases with 60% (12/20), (*P* = 0.011). The proliferation index as detected by Ki-67 ranged from 50–100% (median, 80%), except for the case of MALT lymphoma. BCL2 was positively expressed in 66% (21/32) cases, with a significantly higher frequency among adult patients of 80% (16/20), compared to the pediatric cases of 42% (5/12), (*P* = 0.027). The *IGH-BCL*2 rearrangement was neither detectable among the 10 pediatric cases nor the 11 adult cases with the available data, as it was not evaluated in the rest of the cases. *MYC* breaks were positively expressed in 27.3% (6/22) of our series, and were significantly more frequent among the pediatric cases; constituting half of the 10 evaluated cases and representing all the evaluated BL cases. On the other hand, 8.3% (1/12) adult patient of DLBCL subtype expressed *MYC* breaks (Figure 3).

**Table 2:** Immunophenotypic characteristics of 33 cases with B-cell lymphoma involving Waldeyer's ring

Case no.			IHC				FISH	
	CD10	BCL6	MUM1	Ki−67	BCL2	MYC	IGH/BCL2	MYC
**1−BL**								
1	+	+	−	95%+	−	+	−	+
2	+	+	−	90%+	−	+	−	+
3	+	+	−	100%+	−	+	−	+
4	+	+	−	95%+	−	+	−	+
5	+	ND	ND	95%+	−	ND	ND	ND
6	+	−	−	100%+	−	+	−	+
7	+	+	−	95%+	−	ND	ND	ND
**2−DLBCL**								
PDLBCL								
8	−	+	−	60%+	+	−	−	−
9	+	+	−	80%+	+	−	−	−
10	+	+	−	90%+	+	−	−	−
ADLBCL								
11	+	+	−	80%+	+	ND	−	−
12	−	+	+	80%+	+	−	ND	ND
13	−	+	−	70%+	+	+	−	−
14	−	−	+	90%+	+	ND	−	−
15	−	−	−	70%+	−	ND	ND	ND
17	−	+	+	90%+	+	ND	−	+
16*	+	+	+	90%+	−	−	ND	−
18	−	+	+	70%+	+	ND	−	−
19	+	+	−	70%+	+	ND	ND	ND
20	−	ND	ND	90%+	ND	ND	ND	ND
21	−	+	+	70%+	+	ND	−	−
22	−	+	−	70%+	+	ND	ND	ND
23	−	+	−	80%+	+	−	−	−
24	−	+	−	70%+	+	ND	ND	ND
25*	+	+	+	80%+	+	−	ND	ND
26	+	−	+	60%+	+	ND	ND	ND
27	+	−	+	95%+	−	−	−	−
28	−	+	+	80%+	+	−	−	−
**3− Follicular lymphoma**								
PFL (MUM1 positive lymphoma)								
29	+	+	+	70%+	+	−	−	−
30	+	+	+	50%+	+	−	−	−
AFL / FL 3B								
31	+	+	−	50%+	+	ND	−	−
32	+	+	−	95%+	−	−	−	−
**4−MALT lymphoma**								
33	−	−	−	10%+	+	ND	ND	ND

* In accordance to new classification “large B-cell lymphomas with *IRF*4 rearrangement”.

ADLBCL, adult diffuse large B-cell lymphoma; AFL, adult follicular lymphoma; BL, Burkitt lymphoma; DLBCL, diffuse large B-cell lymphoma; FL, follicular lymphoma; FISH, fluorescence *in situ* hybridization; IHC, immunohistochemistry; MALT, mucosa associated lymphoid tissue; ND, not done; PDLBCL, pediatric DLBCL; PFL, pediatric follicular lymphoma.

Pathological distribution showed that the most common subtypes among the 33 cases of B-cell lymphomas were DLBCL; 64% (21/33), followed by BL; 21% (7/33), FL; 12% (4/33), and MALT lymphoma; 3% (1/33). The 12 pediatric cases comprised of BL, PDLBCL and PFL in 7, 3 and 2 cases, respectively, while 21 adult cases were composed of ADLBCL, AFL, and MALT lymphoma in 18, 2 and 1 case, respectively (Table 3). DLBCL was of significant higher proportion among adult cases compared to the pediatric group (*P* < 0.001), whereas BL subtype was exclusively related to pediatric group.

**Table 3:** Comparison of children and adolescent cases (pediatric group) and adult cases (adult group) with high grade B-cell lymphomas involving Waldeyer's ring

Variable	Group 1 (3–19 years) *n* = 12 no. (%)	Group 2 (20–84 years) *n* = 21 no. (%)	*P*-value
Clinical features			
Median age, year (range)	5.5 (3–19)	57 (27–84)	
M: F	9:3	14:7	0.616
Clinical stage			
Stage I	5/12 (42)	1/17 (6)	0.019
Stage II	4/12 (33)	12/17 (71)	0.047
Stage III	3/12 (25)	4/17 (24)	0.927
Histologic classification			
BL	7/12 (58)	0/21 (0)	
DLBCL	3/12 (25)	18/21 (86)	< 0.001
FL	2/12 (17)	2/21 (9)	0.545
MALT lymphoma	0/12 (0)	1/21 (5)	
Immunohistochemical features			
GCB	12/12 (100)	12/20 (60)	0.011
CD10+	11/12 (92)	8/21 (38)	0.003
MUM1+	2/11 (18)	10/20 (50)	0.082
BCL2+	5/12 (42)	16/20 (80)	0.027
Genetic features			
*IGH/BCL*2	0/10 (0)	0/11 (0)	
*MYC breaks*	5/10 (50)	1/12 (8)	0.029
Outcome			
Relapse	1/9 (11)	1/17 (12)	0.634

BL, Burkitt lymphoma; DLBCL, diffuse large B-cell lymphoma; F, female; FL, follicular lymphoma; GCB, germinal center B-cell; M, male; MALT, mucosa associated lymphoid tissue.

### Different histopathological subtypes of B-cell lymphoma involving WR

Regarding BL, the age range was 3–6 years (median, 4) and (M: F = 5:2), included 14% (1/7) with stage I, 43% (3/7) with stage II, and 43% (3/7) with stage III. BL showed positive expression of CD10 in all the 7 cases, and BCL6 in 83% (5/6), whereas negative expression for both MUM1 and BCL2. As shown in Figure 2 (E), nearly 100% of the neoplastic cells were positive for Ki-67.

Comparing PDLBCL to ADLBCL, the 21 patients with DLBCL and DLBCL/FL ranged from 5–84 years old (median, 54) and a gender ratio of (M: F = 15:6), including PDLBCL patients aged 5–19 years (median, 8) with (M: F = 2:1), and 18 ADLBCL patients with an age range of 27–84 years (median, 56) with (M: F = 13:5). As shown in Table 1, PDLBCL exhibited an earlier clinical process compared to ADLBCL. The 3 cases of PDLBCL presented with stage I in 2 cases and with stage II in 1 patient, and all of them were of GCB phenotype. On the other hand, adult cases with available data of clinical stage were presented with stage II and III, in 71% (10/14) and 29% (4/14) cases, respectively. Evaluation of the ADLBCL cases with the known phenotype disclosed that 53% (9/17) were of GCB, and 47% (8/17) were of the non-GCB phenotype.

Comparing PFL to AFL, 4 cases of FL included 2 pediatric cases of 7 and 11-year-old boys (both with stage I and MUM1-positive-FL) and 2 adult patients of 60 and 64-year-old women (both with stage II and MUM1-negative-FL).

One case of MALT lymphoma was diagnosed in a 58-year-old man who presented with stage I disease, with negative CD10, MUM1 and BCL2, suggesting the non-GCB phenotype. In addition, in the latter case, Ki-67 was positive in 10% of the neoplastic cells.

## DISCUSSION

NHLs involving WR are associated with heterogeneous histological distribution occurring in both pediatric and adult patients. Most of the NHLs involving WR were of B-cell origin with distinctive biological and clinical behavior. However, the relationship among clinical features, pathological subtypes and age groups was unclear. In the current study, 33 of 36 NHL cases were of B-cell lymphoma type, and the subtypes varied among pediatric and adult patients. The most common subtype in children was BL, followed by DLBCL and FL whereas DLBCL was predominantly found in adults, followed by FL.

Histological subtype was the most influencing factor for the clinical significance in patients with lymphomas. BL in children and MALT lymphoma in adults generally represented more favorable outcomes than DLBCL. Therefore, the different distribution and morbidity of histological subtypes would be the main contributor for different clinical presentation between pediatric and adult patients. Age was relatively important to affect the clinical outcome for the same histological subtype of lymphoma. Compared to ADLBCL, as shown in our results, PDLBCL exhibited earlier clinical stages and a higher rate of GCB phenotype, with better prognosis.

As previously described, NHL involving WR demonstrated a more favorable outcome for the localized stage disease compared to the nodal counterpart [3]. Similarly, in our series of cases, most of B-cell lymphomas involving WR presented with stage I/II disease and a significantly higher incidence of stage I among pediatric age group compared to adults. Furthermore, despite the relatively high rate of 43% (3/7) of the advanced clinical stage, the prognosis of BL involving WR in our cases, was associated with favorable outcome and low incidence of recurrence 14% (1/7).

Ki-67 index was closely related to the histopathological subtypes which were different in pediatric and adult patients. Despite of nearly 100% positive expression of Ki-67, BL in children showed a sensitive therapeutic effect and good prognosis. Hence, the relativity of Ki-67 expression and the prognostic value were not correlated in the different subtypes and age groups. In the current report, 32 of 33 cases of B-cell lymphomas were of high grade B-cell lymphomas with a Ki-67 of high proliferation index ranging from 50–100%. Of note, higher proliferation index of at least 80% was associated with poor outcome in adult series [10, 11]. On the other hand, Miles et al, reported that pediatric DLBCL was frequently associated with high histological grade and high proliferation index, however, showed a superior prognosis compared to adults [12]. Similarly, in our pediatric BL series, despite of nearly 100% positive expression of Ki-67, the response was good.

The GCB phenotype was reported to occur in 50–60% of adult DLBCL and was associated with a better prognosis compared to those with non-GCB phenotype [13, 14]. Comparable to previous report concerned with PDLBCL, which showed a higher proportion of GCB subtype and a more superior prognosis as compared with adult disease, our data disclosed that 75% of the B-cell lymphoma cases involving WR were of GCB phenotype, representing all the pediatric cases, and 60% of adult cases phenotype [12]. MUM1 represented the most important non-GCB marker, and the clinical meaning of MUM1 and CD10 mainly related to the histological subtypes of lymphomas.

Although BCL2 was positive in 66% of cases, no *IGH*/*BCL*2 rearrangement was detectable in this study, in accordance with previous studies which showed low rates of *BCL2* translocations of DLBCLs involving WR [3]. Taken together, BCL2 protein expression was reported to be associated with poor prognosis, however, recent studies suggested that the prognostic value of BCL2 expression is restricted to non-GCB tumor only [15].

*MYC* rearrangement was identified in 100% (5/5) BL cases and 8% (1/13) case of DLBCL, whereas no *BCL*2 rearrangement was detectable in our series. *MYC* rearrangement was related to poor prognosis in DLBCL and FL, but not in BL. Thus, *BCL*2 and *MYC* rearrangements affect the disease prognosis differently in accordance to the histological subtypes of lymphoma. Moreover, MYC/BCL2 protein co-expression contributes to the inferior survival of non-GCB subtype of DLBCL [16]. Therefore, the low co-expression of BCL2/MYC in our series of cases could explain our observation of the favorable prognosis of B-cell lymphomas involving WR.

As reported recently, *IRF*4 translocation was identified as a primary molecular alteration in a subset of GCB-derived lymphomas. The probability for this subtype of lymphoma significantly decreases with age, suggesting that diversity in tumor biology might contribute to the age-dependent differences in the prognosis of lymphoma [17].

Recent studies highlighted the significance of MUM1 expression in PFL involving WR. Meanwhile, compared to its nodal counterpart, PFL involving WR was thought to be a distinctive subtype of PFL, characterized by the uniform positivity for MUM1 [6]. Though, the incidence of MUM1 expression in both the PDLBCL and the AFL involving WR, had not yet been described.

Our two patients (cases 29 and 30) were initially diagnosed after surgery as PFL grade 3B with MUM1-positive-lymphoma in WR. However, with the updated understanding of WR, these cases would best fit the new entity classification of large B-cell lymphomas with *IRF*4 rearrangement [18]. In contrast, MUM1 expression, was not detectable in other subtypes of our cases of pediatric B-cell lymphoma involving WR, including BL and PDLBCL. Therefore, MUM1-positive expression was the “hall mark” of immunoreaction for PFL involving WR, which might be helpful for distinguishing this newly recognized entity from other pediatric lymphomas. A previously reported paper disclosed that some cases of MUM1-positive-lymphoma expressed CD5 protein as well [6]. Interestingly, one of our patients (case 30) was strongly and diffusely positive for CD5, therefore, we reported the child's clinicopathological presentation in details [19].

## MATERIALS AND METHODS

Our study was approved by the Ethics Committee of Xinhua Hospital Affiliated to Shanghai Jiaotong University School of Medicine. All cases were obtained from the files of a single institution. This study was based on a retrospective review of 443 cases previously diagnosed as lymphoma within the period from September 2012 to January 2016. Among the total of 443, 36 cases of lymphomas involving WR were identified according to histological and immunohistochemical analyses, in reference to 2008 World Health Organization classification. Ann Arbor classification was used for staging. Clinical information was recorded for each patient including age, gender, date of initial diagnosis, symptoms and signs at presentation, clinical and/or pathological stage, in addition to treatment information. Patient's survival status and follow-up time were assessed as well.

Nine pediatric patients received chemotherapy in accordance to the China Children's Cancer Group (CCCG)-BNHL protocol. On the other hand, the protocols used for adults included either R-CHOP (rituximab, cyclophosphamide, adriamycin, vincristine, and prednisone), or R-EPOCH (rituximab, etoposide, prednisone, vincristine, cyclophosphamide, and adriamycin), or both R-CHOP and R-EPOCH regimens were used in 10, 3, and 3 patients, respectively. The patient with MALT lymphoma received radiation only. Written informed consent was previously obtained from each patient and/or guardians in accordance to the guidelines of the Declaration of Helsinki.

### Histologic and immunohistochemistry analyses

Formalin-fixed, paraffin-embedded tissue samples were available for all the 36 cases and were stained with hematoxylin and eosin (HE) at initial diagnosis. Immunohistochemistry was performed using a panel of monoclonal and polyclonal antibodies, as follows: CD20 (clone L26, DAKO, Glostrup, Denmark); CD79a (clone 1.10E+04, Leica Biosystems, Wetzlar, Germany); PAX5 (clone R1, DAKO); CD10 (clone 56C6, Leica Biosystems); BCL6 (clone P1F6, DAKO); MUM1 (clone MUM1p, DAKO); Ki-67 (clone MIB-1, DAKO); BCL2 (clone 100/D5, DAKO); MYC (DAKO); EBV (DAKO); terminal deoxynucleotidyl transferase (TDT, clone SP150, DAKO); CD3 (clone LN10, Leica Biosystems); CD43 (clone DF-T1, DAKO). MYC was considered as positive when > 40% of tumor cells exhibited staining [20]. Positivity threshold was defined at 50% for BCL2 [20], and at 30% for CD10, BCL6, and MUM1 [4]. For sub-classification of DLBCLs, GC immunophenotype was evaluated by antibodies of C10, BCL6 and MUM1 [4].

### FISH analyses

FISH was performed on 3–4 μm thick sections of formalin-fixed paraffin-embedded tissue samples, employing *BCL*2 (LSI *IGH/BCL2* dual-color dual-fusion probe, Vysis-Abbott, IL, USA), and *MYC* (LSI*MYC* Dual Color, BreakApart Rearrangement Probe, Vysis-Abbott, IL, USA) to detect t(14;18)(q32;q21) (*IGH/BCL*2) and *MYC* gene rearrangement, respectively. The cut-off values for the interphase FISH analyses were established following the criteria of Ventura et al, and for each sample 100 evaluable nuclei with complete FISH signals were scored [21].

### Statistical analysis

Contingency tables were analyzed using Pearson chi-square statistic. Differences with *P*-value < 0.05 were defined as statistical significance. The software of SPSS version 17.0 (SPSS Inc., Chicago, IL, USA) was used for statistical calculations.

## CONCLUSIONS

In conclusion, B-cell lymphomas involving WR presented with a wide age range, male predominance, high rate of the localized clinical stage, a favorable outcome despite the high-grade histopathology, high rate of GCB phenotype, and more frequency of BCL2 expression lacking *IGH/BCL*2 rearrangement. Moreover, pediatric patients differed from adults in terms of clinical features, histopathological subtypes and immunophenotypes. Notably, MUM1 was positive in a subset of GCB-ADLBCL and exclusively in MUM1-positive-lymphoma/PFL involving WR among pediatric patients.
